# Computational repositioning of dimethyl fumarate for treating alcoholic liver disease

**DOI:** 10.1038/s41419-020-02890-3

**Published:** 2020-08-18

**Authors:** Ye Zhang, Shuang Zhao, Ying Fu, Lu Yan, Yilu Feng, Yaqi Chen, Yijia Wu, Yalan Deng, Guiying Zhang, Zhuchu Chen, Yongheng Chen, Ting Liu

**Affiliations:** 1 Department of Oncology, NHC Key Laboratory of Cancer Proteomics, State Local Joint Engineering Laboratory for Anticancer Drugs, National Center for Geriatrics Clinical Research, Xiangya Hospital, Central South University, 410008 Changsha, Hunan China; 2 Department of Gastroenterology, Xiangya Hospital, Central South University, 410008 Changsha, Hunan China

**Keywords:** Enzyme mechanisms, Mechanisms of disease

## Abstract

Alcoholic liver disease (ALD) is a chronic alcohol-induced disorder of the liver for which there are few effective therapies for severe forms of ALD and for those who do not achieve alcohol abstinence. In this study, we used a systematic drug-repositioning bioinformatics approach querying a large compendium of gene-expression profiles to identify candidate U.S. Food and Drug Administration (FDA)–approved drugs to treat ALD. One of the top compounds predicted to be therapeutic for ALD by our approach was dimethyl fumarate (DMF), an nuclear factor erythroid 2-related factor 2 (NRF2) inducer. We experimentally validated DMF in liver cells and in vivo. Our work demonstrates that DMF is able to significantly upregulate the NRF2 protein level, increase NRF2 phosphorylation, and promote NRF2 nuclear localization in liver cells. DMF also reduced the reactive oxygen species (ROS) level, lipid peroxidation, and ferroptosis. Furthermore, DMF treatment could prevent ethanol-induced liver injury in ALD mice. Our results provide evidence that DMF might serve as a therapeutic option for ALD in humans, and support the use of computational repositioning to discover therapeutic options for ALD.

## Introduction

Oxidative stress is implicated in the development of diverse liver disorders, such as alcoholic liver disease (ALD)^1,2^. ALD encompasses a variety of chronic liver diseases, including liver steatosis (fatty liver), hepatitis (combined with inflammation), fibrosis, cirrhosis, and ultimately hepatocellular carcinoma (HCC)^3^. Although alcohol abstinence is effective for patients with mild ALD (steatosis), there are few effective therapies for severe forms of ALD and for those who do not achieve alcohol abstinence. Corticosteroid is the only treatment option to improve the short-term survival of severe alcoholic hepatitis (AH) patients^4^. However, many of these patients do not respond to this treatment, and experience severe adverse effects, such as infection^5^. Therefore, there is an urgent need to develop novel targeted therapeutics to treat severe forms of ALD or patients who fail to achieve alcohol abstinence. The computational repositioning of Food and Drug Administration (FDA)-approved drugs is a promising and efficient avenue for discovering new uses^6^. Given the high costs, possible side effects, high failure rate, and long testing periods for developing new medicines, an FDA-approved compound was known to be generally safe in humans and available for clinical use^7^. It is possible to identify safe drugs with potential for repurposing in other conditions by using computational strategies, which can eliminate the need for a Phase I safety trial and expedite Phase II efficacy trials. Analysis of interactions between genes and FDA-approved drugs allow the pursuit of new indications for treating diseases with no FDA-approved pharmacotherapies.

Recent advancements in computing and the dramatic expansion of available high-throughput datasets have enabled the development of drug repurposing to identify novel treatment options for ALD. Thus in this study, we aimed to identify a new therapeutic option with potential for repositioning in ALD. We used a systematic computational approach based on both public gene-expression patterns in ALD and the interactions between genes and FDA-approved drugs. Interestingly, we identified nuclear factor erythroid 2-related factor 2 (NRF2) as a novel therapeutic target in ALD^8^. NRF2 is a basic leucine zipper (bZIP) transcription factor that regulates the expression of certain proteins, which protect cells against oxidative stress. Under unstressed conditions, NRF2 is kept in the cytoplasm by Kelch like-ECH-associated protein 1 (KEAP1) and Cullin3. Upon oxidative stress, NRF2 is phosphorylated at Ser40 and releases from KEAP1, then translocates into the nucleus. In the nucleus, NRF2 forms a heterodimer with one of the small MAF proteins (MAFF, MAFG, and MAFK), binds to the antioxidant response element (ARE) in the promoter regions of many antioxidative enzymes, and regulates the transcription of these enzymes, such as glutamate–cysteine ligase, catalytic (GCLC) and heme oxygenase-1 (HO1). More surprisingly, we found that the FDA-approved NRF2 inducer^9^, dimethyl fumarate (DMF), which has not previously been described to have a therapeutic association with ALD, was determined to have a strong therapeutic potential for repositioning in ALD. We evaluated the efficacy of DMF for ALD in liver cells and in vivo, using an ethanol-induced mouse model. Concordant with our computational prediction, the experimental results demonstrate that DMF is able to significantly ameliorate ethanol-induced liver injury compared to untreated groups.

## Results

### Computational repositioning of FDA-approved drugs for ALD

**Fig. 1:**
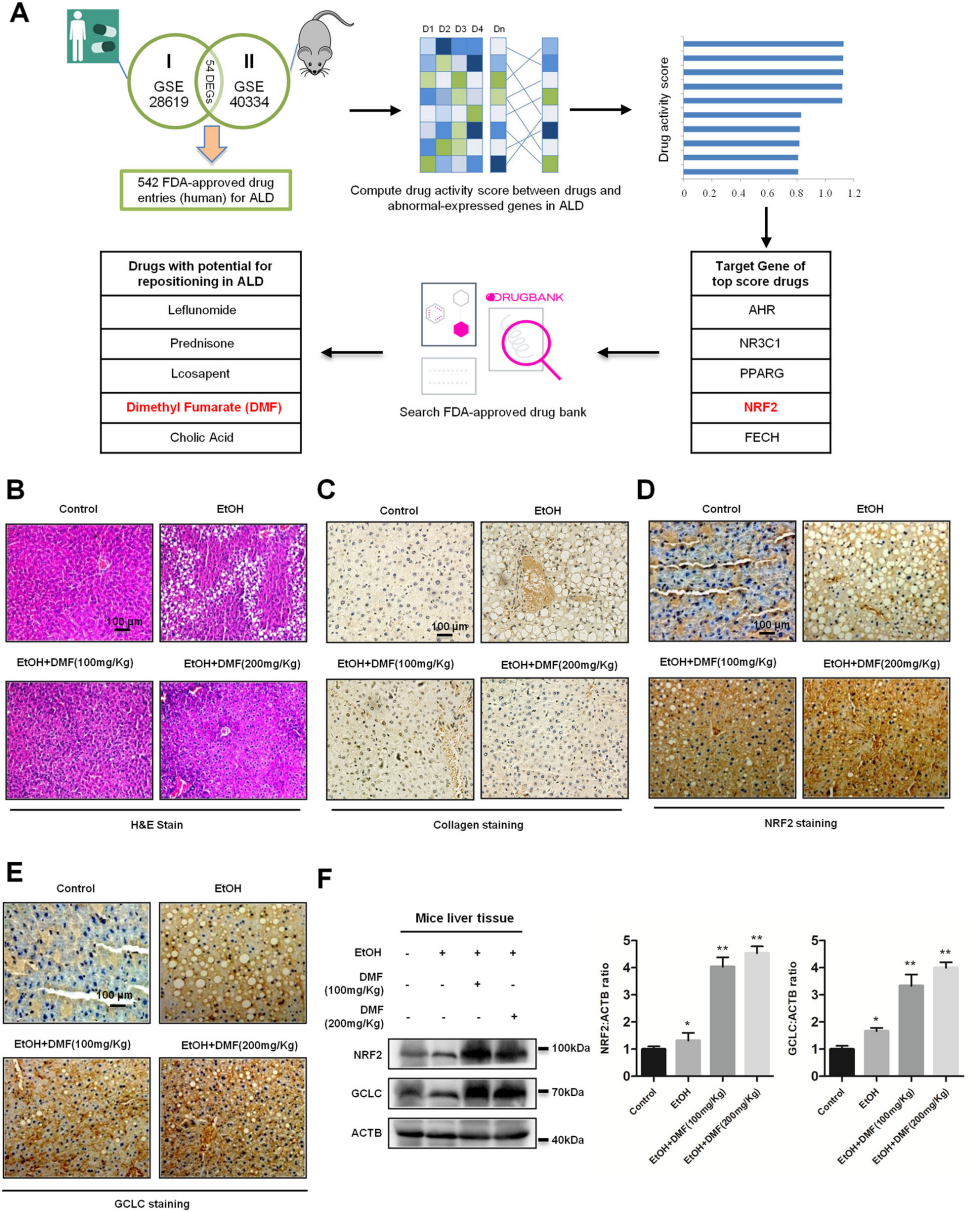
Computational repositioning of Food and Drug Administration (FDA)-approved drugs for alcoholic liver disease (ALD). **a** Schematic representation of the bioinformatics workflow for the repositioning approach used to identify potential candidate drugs and genes for the treatment of ALD. **b** Dimethyl fumarate (DMF) prevents ethanol-induced hepatic steatosis. Mice were fed with the control diet or ethanol diet containing 5% (v/v) ethanol, respectively, followed by treatment with 100 mg/kg DMF or 200 mg/kg DMF by oral gavage for 10 days. Tissue sections from the mouse liver were prepared for hematoxylin and eosin (H&E) staining. Scale bars are 100 μm. **c** DMF decreases ethanol-induced hepatic fibrosis. Mice were fed as in (**b**), tissue sections from the mouse liver were prepared for collagen staining. Scale bars are 100 μm. **d**, **e** DMF increases endogenous NRF2 and GCLC to activate the NRF2 signaling pathway in the mouse liver. Immunohistochemical staining of NRF2 and GCLC proteins in mouse liver tissues. Liver tissue sections from different groups were stained immunohistochemically with anti-NRF2 antibody (**d**) or anti-GCLC antibody (**e**) as indicated. Data shown are from one mouse from each group. Scale bars are 100 μm. **f** NRF2 and GCLC in mouse liver sections were compared against ACTB by western blotting. The statistical analysis of all samples is shown.

To identify efficient therapeutic strategies for patients with liver diseases, we downloaded drug datasets that contain both clinical application and animal test from Gene Expression Omnibus (www.ncbi.nlm.nih.gov/geo/GSE accession number GSE28619 and 40334). Then we used a bioinformatics approach to test the drug-repositioning potential of FDA-approved drugs for ALD. From this approach, we computed the activity score of candidate drugs and compared gene-expression profiles in response to these drugs in ALD. Then we annotated the known gene targets of the top-scoring candidates, and queried FDA-approved DrugBank using gene targets as an input, which displayed an output of a list of chemical compounds. Notably, ALD cells are known to abnormally express molecules in the antioxidant response pathway, thus we aimed to study one of the five top-scored candidate genes, NRF2. Among NRF2-compound interactions, the main use of DMF is previously tested with some success in multiple sclerosis patients with relapsing forms, suggesting that DMF used in the clinic may affect the ALD gene-expression signature. This analysis led us to focus on drugs targeting molecules (Fig. 1a). The majority of known physiologic or pharmacological NRF2 inducers are electrophilic molecules that covalently modify, by oxidation or alkylation, cysteine residues present in the thiol-rich KEAP1 protein^10^. DMF is one of the known NRF2 inducers, which has been tested for the treatment of multiple sclerosis, and approved in 2013 for its drug bioavailability and efficacy^11^. Currently, MMF has been used to develop a second generation of NRF2 inducers as prodrugs^12^. Therefore, we focused on the fumarate-regulation mechanism of NRF2 in liver disorders. The generation of toxic metabolites by ethanol, such as lipid-peroxidation products, contributes to the pathogenesis of alcoholic liver injury. Fumarates prevent ROS accumulation via the NRF2 pathway in liver cells. Therefore, we used an ALD mouse model (six mice a group) and hepatic fibrosis rat model (nine rats a group) to examine the role of fumarates in vivo. Hepatic lipid accumulation was distinctively increased in ethanol-fed rats. In order to address the role of DMF in hepatic lipid accumulation, we administered ALD mice with DMF at 100 mg/kg/day or 200 mg/kg/day for 10 days. In order to address the role of DMF in hepatic fibrosis, we administered hepatic fibrosis rats with DMF at 15 mg/kg/day or 25 mg/kg/day for 8 weeks. DMF ameliorated the hepatic steatosis induced by ethanol, as observed in liver sections stained with hematoxylin and eosin (H&E) (Fig. 1b and Supplementary Fig. S1A). At the same time, the highly cross-linked collagen fraction increased significantly during ethanol-induced fibrosis progression, while collagen deposition was partly reduced under DMF treatment (Fig. 1c and Supplementary Fig. S1B). To substantiate the finding that DMF increases the activity of NRF2 pathway to inhibit ALD, we collected liver sections from normal and ALD mice and checked NRF2 and GCLC protein levels in the mouse model. We performed immunohistochemistry (IHC) and western blotting for NRF2 and GCLC. Results revealed that DMF treatment significantly increased NRF2 and GCLC protein levels in ALD mouse liver, when compared to the matched control groups (Fig. 1d–f and Supplementary Fig. S1C, D).

### DMF and MMF activate the NRF2 pathway in liver cells

**Fig. 2:**
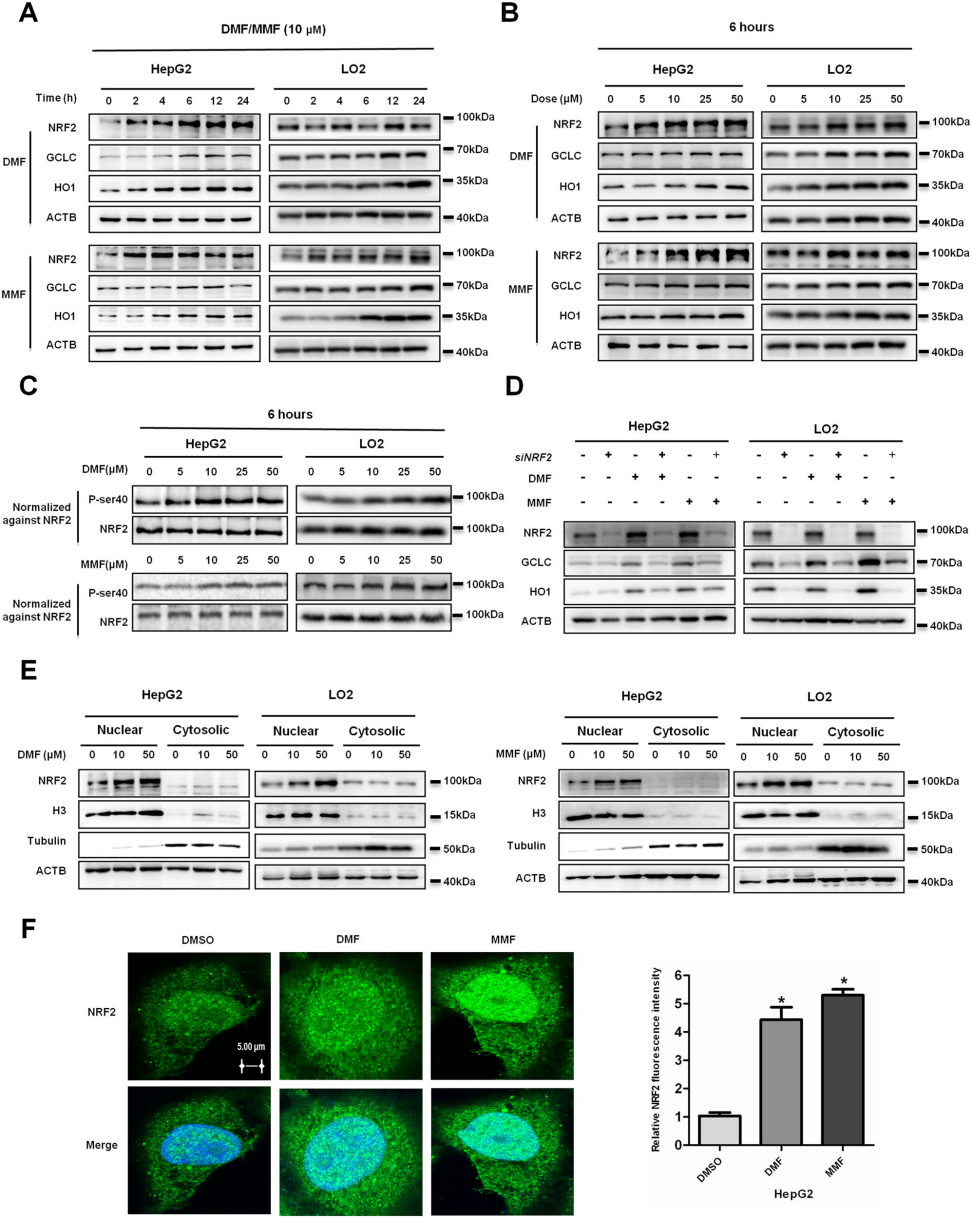
Dimethyl fumarate (DMF) and MMF activate the NRF2 pathway in liver cells. **a** DMF or MMF treatment increases endogenous NRF2, GCLC, and HO1 protein level in a time-dependent manner. HepG2 or LO2 cells were either untreated or treated with 10 μM DMF or MMF for different lengths of time, followed by being lysed and subjected to western blotting with the indicated antibodies. **b** DMF or MMF treatment increases endogenous NRF2, GCLC, and HO1 protein level in a dose-dependent manner. HepG2 or LO2 cells were either untreated or treated with DMF or MMF at the indicated concentrations for 6 h. ACTB is shown as a loading control. **c** DMF or MMF increases the NRF2 S40 phosphorylation level. HepG2 or LO2 cells were treated as in (**b**), analyzed by western blotting with NRF2 (phospho S40) antibody, and normalized against NRF2 protein (The sample loading was adjusted to keep the NRF2 level constant). **d** *NRF2* knockdown decreases GCLC and HO1 protein levels under normal or fumarates condition. HepG2 or LO2 cells were transfected with si*NRF2* or negative control. NRF2, GCLC, and HO1 protein levels were determined by western blotting. **e** DMF or MMF promotes NRF2 nuclear accumulation. After treated with 10 μM DMF (left panel) or MMF (right pannel) for 6 h, HepG2 or LO2 cells were subjected to cytosolic and nuclear fractionation, and NRF2 protein levels were determined by western blotting. Histone-3 (H3) and α-tubulin were used as nuclear and cytoplasmic markers, respectively, while ACTB was used as a whole-cell lysate maker. **f** HepG2 cells were treated with DMSO, 10 μM DMF, or 10 μM MMF for 6 h as indicated, then paraformaldehyde fixed, blocked, and processed for immunofluorescence with DAPI (blue) or antibody against NRF2 (green). NRF2 staining is shown on the left and the merged NRF2 and DAPI on the right. Bar: 5 μm. Relative NRF2 fluorescence intensity was calculated using ImageJ software; the ratio was quantified. Mean values were calculated from the individual distributions in ten cells per condition.

NRF2 is an essential regulator of the antioxidant response pathway, which promotes the expression of various genes in response to oxidative stress^13,14^. Fumarates protect neurons and astrocytes against ROS damage^15^. To determine whether DMF or MMF regulates the NRF2 protein level in liver cells, we cultured HepG2 and LO2 cells under the treatment of 10 μM DMF and MMF for different lengths of time, and found that both DMF and MMF increased the protein level of NRF2 in a time-dependent manner (Fig. 2a and Supplementary Fig. S2A). Further results revealed that the NRF2 protein level was upregulated with increased DMF and MMF concentrations (Fig. 2b and Supplementary Fig. S2B). Phosphorylation serine-40 is required for NRF2 activation^16,17^. To confirm the activation of NRF2, we treated HepG2 or LO2 cells with DMF and MMF, respectively, as indicated, then determined the level of phosphorylated NRF2 protein by western blotting. Results showed that DMF and MMF treatment significantly increased the phosphorylation level of NRF2 when we adjusted the sample loading to keep the NRF2 level constant (Fig. 2c and Supplementary Fig. S2C), indicating that NRF2 was activated. In addition, we checked the protein levels of NRF2-regulated genes^15^. Our data showed that DMF and MMF treatment promoted the expression of GCLC and HO1 protein levels (Fig. 2a, b). Moreover, *NRF2* knockdown dramatically decreased GCLC and HO1 protein upon either normal condition or fumarates treatment (Fig. 2d and Supplementary Fig. S2D). Collectively, our results demonstrate that fumarates activate the NRF2 pathway in liver cells.

Once phosphorylated, NRF2 can translocate into the nucleus, and activate transcription of various detoxification and antioxidant enzymes upon exposure to stresses^18^. To examine whether fumarates regulated NRF2 nuclear localization in liver cells, we treated HepG2 or LO2 cells with DMF and MMF at different concentrations for 6 h (Fig. 2e). Then cells were lysed and subjected to cytosolic and nuclear fraction extraction. We found that DMF (Fig. 2e, left pannel) and MMF (Fig. 2e, right pannel) promoted NRF2 nuclear accumulation in a dose-dependent manner. Moreover, we performed immunofluorescence in liver cells. Confocal microscopy data showed that NRF2 expression and nuclear localization were enhanced in HepG2 cells upon DMF and MMF treatment (Fig. 2f). Taken together, our data provide evidence that fumarates activate NRF2 and promote its translocation from cytoplasm to the nucleus.

### DMF and MMF reduce the ROS level by activating NRF2 in liver cells

**Fig. 3:**
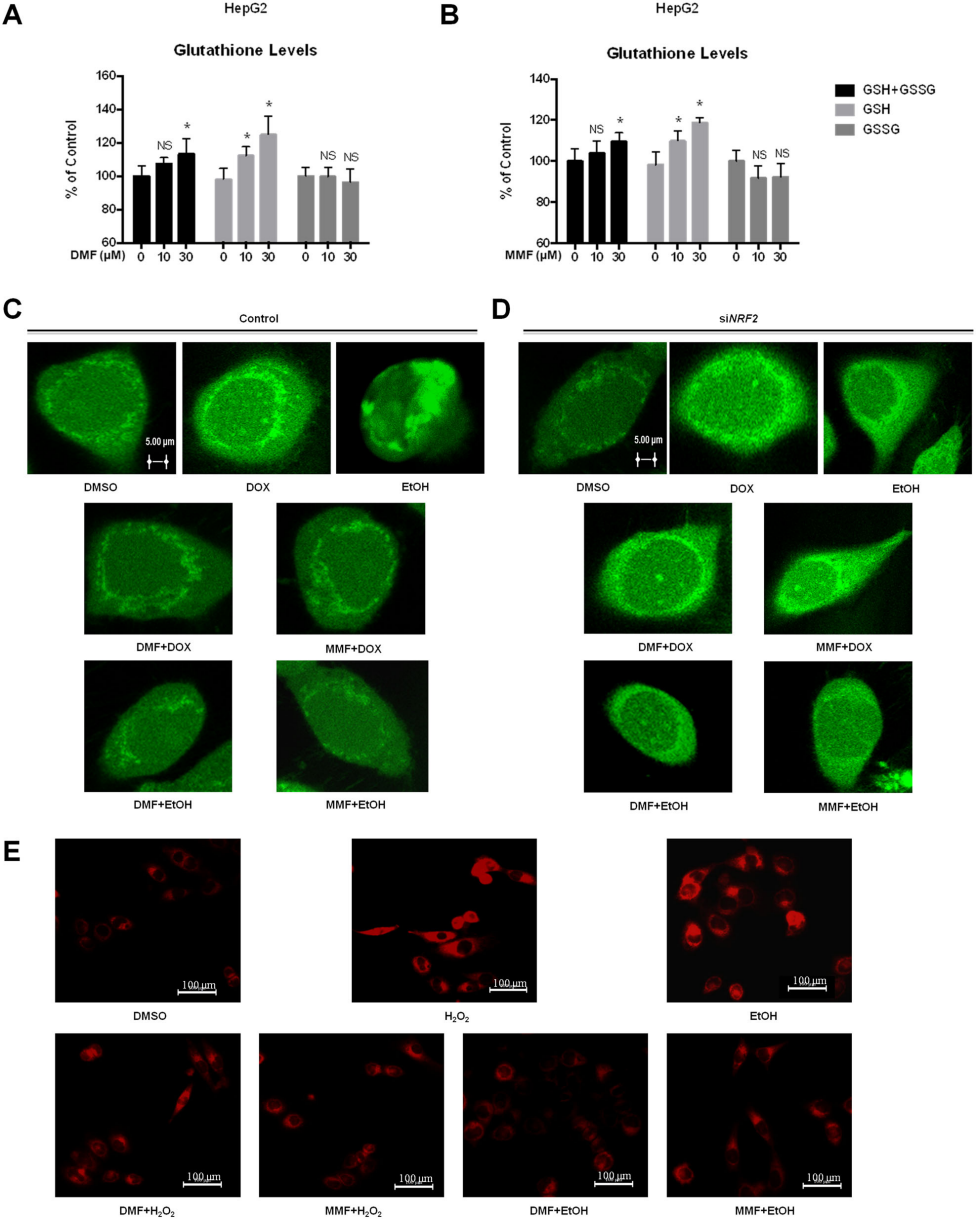
Dimethyl fumarate (DMF) and MMF reduce the ROS level by activating NRF2 in liver cells. **a**, **b** DMF or MMF enhances cellular redox potential by increasing GSH level. HepG2 cells were treated with or without different concentrations of DMF (**a**) or MMF (**b**) for 20 h, and then assessed for cellular GSH and GSSG levels. *Denotes *P* < 0.05, NS denotes no significance. Error bars represent mean ± SD for triplicate experiments. **c** Fumarates block DOX or ethanol-induced ROS accumulation. HepG2 cells were pre-treated with DOX or ethanol for 6 h, followed by treatment with 10 μM DMF or MMF for another 6 h as indicated. Cells were loaded with DCFH-DA (10 μM) and incubated for 30 min at 37 °C in the dark. Fluorescence images were acquired by a confocal microscope. Bar: 5 μm. **d** *NRF2* knockdown accumulates ROS damage in liver cells, either with or without fumarates. HepG2 cells were transfected with si*NRF2* and treated as in (**c**). Fluorescence images were obtained. **e** Fumarates block H_2_O_2_ or ethanol-induced mitochondrial ROS accumulation. LO2 cells were pre-treated with H_2_O_2_ or ethanol for 6 h, followed by the treatment with 10 μM DMF or MMF for another 6 h as indicated. Cells were incubated with MitoTracker^®^ Red CMXRos (red) at 37 °C in the dark. Images were acquired by fluorescence microscope. Bar: 100 μm.

The relative levels of GSH and GSSG are associated with various disease, aging, and cell signaling events^19–21^. To illustrate the potency of fumarates as antioxidant agents, we performed the reaction to convert total glutathione and the oxidized form (GSSG) to the reduced form (GSH). Then we measured both total glutathione and GSSG in the luminescent reaction scheme with the GSH probe. The results showed that DMF and MMF induced a dose-dependent increase of intracellular GSH (Fig. 3a, b). Doxorubicin (DOX), an effective anticancer agent, can induce the generation of ROS, which then leads to oxidative damage of cellular and mitochondrial membranes^22,23^. 2-7-Dichlorofluorescin diacetate (DCFH-DA) is a specific indicator of ROS formation^24^ and has been used widely as a fluorescence probe in cells^25,26^. Confocal microscopy data revealed that ROS were accumulated in HepG2 cells with the presence of DOX, while DMF and MMF blocked the DOX-induced accumulation of ROS (Fig. 3c and Supplementary Fig. S3A). Then we performed siRNA transfection in HepG2 cells to knock down *NRF2*, and observed a significant increase of ROS upon DOX treatment, even in the presence of DMF and MMF (Fig. 3d and Supplementary Fig. S3B). Moreover, we used MitoTracker^®^ Red CMXRos Kit, an agent which can be passively transported through the cell membrane and directly gathered on the active mitochondria, to test the effect of fumarates on the mitochondrial ROS level. We found a significant reduction of H_2_O_2_ or ethanol-induced mitochondrial ROS under fumarates treatment (Fig. 3e and Supplementary Fig. S3C).These results suggest a resistant effect of fumarates in response to ROS by activating the NRF2 pathway.

### DMF and MMF reduce ROS-induced lipid peroxidation and ferroptosis in liver cells

**Fig. 4:**
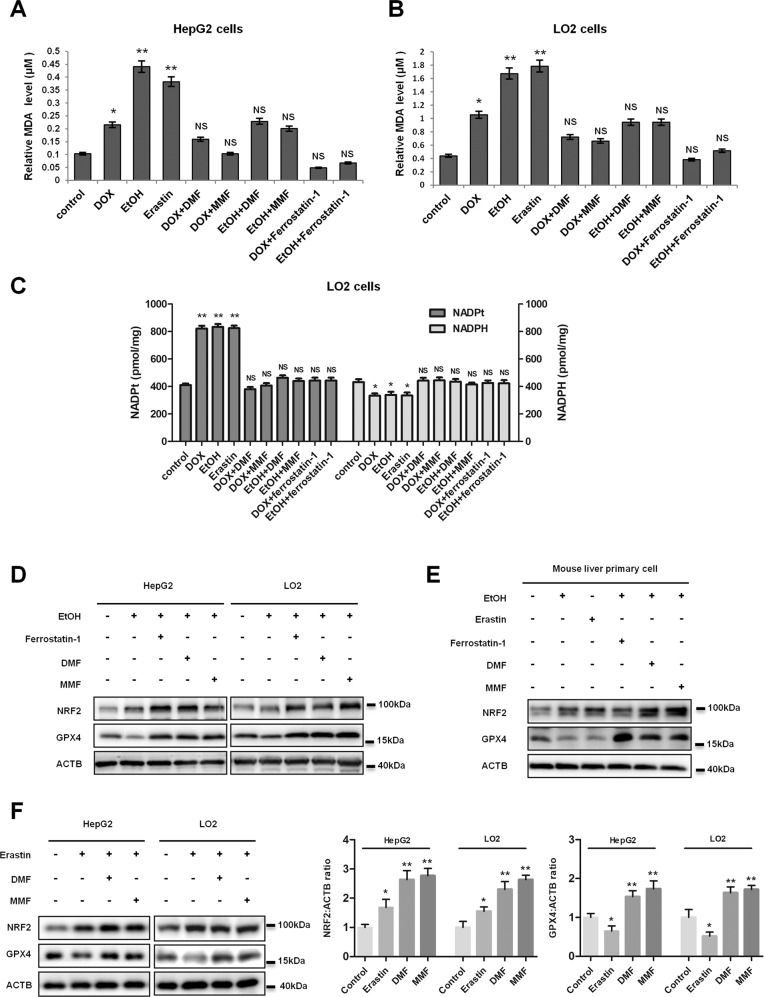
Dimethyl fumarate (DMF) and MMF reduce ROS-induced lipid peroxidation and ferroptosis in liver cells. **a**, **b** Fumarates obviously reverse DOX or ethanol-induced lipid peroxidation. HepG2 (**a**) and LO2 (**b**) cells were pre-treated with 10 μM DOX, 80 mM ethanol, or 10 μM erastin for 6 h, followed by 1 μM ferrostatin-1 or 10 μM fumarates for 6 h. Thereafter, cells were lysed and subjected to lipid peroxidation (malondialdehyde, MDA) assay. **c** Fumarates reverse DOX or ethanol-induced ferroptosis. LO2 cells were pre-treated with 10 μM DOX, 80 mM ethanol, or 10 μM erastin for 6 h, followed by 1 μM ferrostatin-1 or 10 μM fumarates for 6 h. Thereafter, cells were lysed and subjected to NADP/NADPH assay. *Denotes *P* < 0.05, ** denotes *P* < 0.01, and NS denotes no significance. Error bars represent mean ± SD for triplicate experiments. **d**–**f** Fumarates block lipid peroxidation and ferroptosis in liver cells. HepG2 cells, LO2 cells (**d**, **f**), or mouse liver primary cells (**e**) were pre-treated with 80 mM ethanol or 10 μM erastin for 6 h as indicated, followed by 1 μM ferrostatin-1 or 10 μM fumarates for another 6 h. Thereafter, cells were lysed and subjected to western blotting for NRF2 and GXP4, with ACTB as loading control. The statistical analysis of all samples is shown (**f**).

Recent studies showed accumulation of ROS can lead to lipid peroxidation and ferroptosis^27^, therefore, we speculated that fumarates regulate ROS-induced ferroptosis. To examine ferroptosis in DOX or ethanol-treated cells, we examined the levels of hepatic malondialdehyde (MDA) and NADP/NADPH content^28,29^. Consistent with ROS-induced ferroptosis, we found that DOX or ethanol treatment significantly increased lipid peroxidation (Fig. 4a, b), and decreased NADPH content (Fig. 4c). We observed a decrease of MDA levels and a restoration of NADPH, when we added fumarates into liver cells pre-treated with DOX or ethanol (Fig. 4a–c). More evidence was obtained when we detected the protein level of GPX4, an important ferroptosis regulator, which can inhibit cell membrane phospholipid peroxidation. Results showed that compared with DMSO treatment, GXP4 was substantially decreased under ethanol-stimulated condition, indicating a promoting role of ethanol in liver lipid peroxidation and ferroptosis. However, we observed a restoration of the GXP4 protein level, when we added ferrostatin-1 (an inhibitor of ferroptosis) into HepG2 and LO2 cells pre-treated with ethanol (Fig. 4d and Supplementary Fig. S4A). A similar result was detected in mouse liver primary cells. Ethanol treatment lead to a significant decrease of endogenous GPX4, while ferrostatin-1 restored GPX4 protein pre-treated with ethanol (Fig. 4e and Supplementary Fig. S4B). In addition, we treated liver cells with erastin, an inducer of ferroptosis, which plays the opposite role to ferrostatin-1 in ferroptosis, and found fumarates led to an accumulation of GXP4 and NRF2 protein even in the presence of ethanol or erastin (Fig. 4e, f). We also detected lipid peroxidation with C11-Bodipy 581/591 undecanoic acid, by measuring the fluorescence intensity in red color. Consistent with our previous results, an increase of ROS production was observed under the treatment of ethanol and erastin, while ferrostatin-1 or fumarates can inhibit lipid peroxidation induced by ethanol (Supplementary Fig. S4C), suggesting a preventive effect of fumarates in ROS-induced lipid peroxidation and ferroptosis.

### DMF inhibits ethanol-induced lipid peroxidation and ferroptosis in vivo

**Fig. 5:**
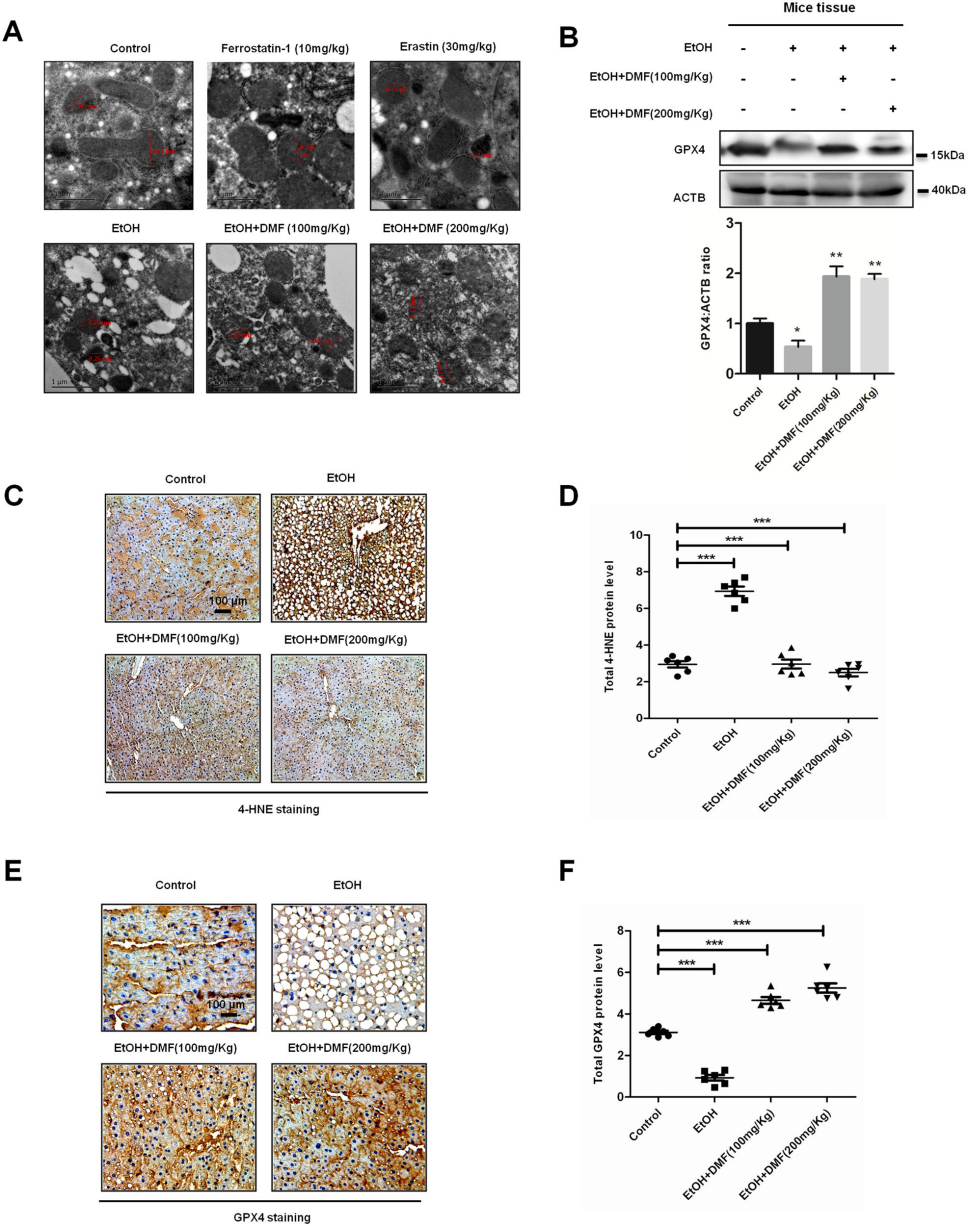
Dimethyl fumarate (DMF) inhibits ethanol-induced lipid peroxidation and ferroptosis in vivo. **a** DMF prevents ethanol-induced ferroptosis. Mice were fed as indicated. On the final day morning, the mice were given alcohol liquid (5 g/kg) or maltodextrin (control) by gavage, and sacrificed after 9 h. In addition, ferrostatin-1 (10 mg/kg) and erastin (30 mg/kg) were provided 15 min before gavage by intraperitoneal injection. Tissue sections from the mouse liver were prepared for electron microscopy observation. Scale bars are 100 μm. **b** GPX4 protein in mouse liver sections was examined by western blot with ACTB as a loading control. **c**, **e** DMF inhibits ethanol-induced lipid peroxidation and upregulates GPX4 protein to inhibit ferroptosis in vivo. Mice were treated as indicated with ethanol and DMF. Liver tissue sections from different groups were stained immunohistochemically with anti-HNE4 antibody (**c**) or anti-GPX4 antibody (**e**) as indicated. Data shown are from one mouse from each group. Scale bars are 100 μm. **d**, **f** The statistical analysis of all samples is shown.

These results strongly suggest that DMF prevents ROS-induced liver injury and ferroptosis via activating the NRF2 pathway. We therefore studied the role of DMF in ROS-induced ferroptosis in mice hepatocytes treated with ethanol or not. Compared to the untreated group and ferrostatin-1 treated group, groups treated by ferroptosis-inducer erastin and ethanol had smaller, ruptured mitochondria (Fig. 5a); these cellular morphological features are characteristic of ferroptosis. However, DMF ameliorated the ferroptosis induced by ethanol, as observed by transmission electron microscopy.

More evidence was obtained when we performed western blotting and IHC. Compared with the normal mice, the protein levels of 4-HNE, which indicated an increased lipid-peroxidation-induced ferroptosis, were higher in ALD mouse livers, while the GPX4 protein level was lower. In contrast, DMF treatment could block lipid-peroxidation-induced ferroptosis by decreasing the protein levels of 4-HNE and increasing the protein levels of GPX4 in vivo (Fig. 5b–f). These data further validate fumarates as inhibitors of the lipid-peroxidation-induced ferroptosis.

## Discussion

Using a computational repositioning of existing drugs based on the publicly available gene-expression data to discover therapies for ALD, we inferred that the NRF2 inducer DMF could serve as a therapeutic option for ALD, and performed experimental validations which demonstrated the efficacy of DMF in ameliorating ALD in liver cells and in the mouse model. The precise mechanism of action for DMF is unknown, but it is known to activate the NRF2 antioxidant pathway. Although DMF has not previously been suggested as a therapy for ALD, previous study has shown that NRF2 prevents alcohol-induced fulminant liver injury^30^. In this study, we found that fumarates activate the NRF2 signaling pathway, promoting NRF2 phosphorylation and nuclear localization in liver cells. NRF2 further activates the transcription of genes encoding various detoxification and antioxidant enzymes in response to ROS.

Oxidative stress is implicated in the development of diverse liver disorders, such as ALD, nonalcoholic fatty liver disease (NAFLD), and HCC^2^. Elevated cellular stresses, which are induced by alcohol, hepatic viruses, or drugs, play a vital role in the initiation and progression of multiple liver pathologies^31–33^. Certain stressed conditions can cause the accumulation of cellular ROS. Uncontrolled production of ROS results in oxidative stress on tissues and cells and causes lipid peroxidation^34^. The NRF2 antioxidant pathway is a highly conserved signal transduction pathway that allows cells, tissues, and organs to survive under oxidative stress conditions^35^. Our study showed that fumarates activate the NRF2 signaling pathway, reduce the cellular ROS level, and protect liver cells from ethanol-induced oxidative injury.

**Fig. 6:**
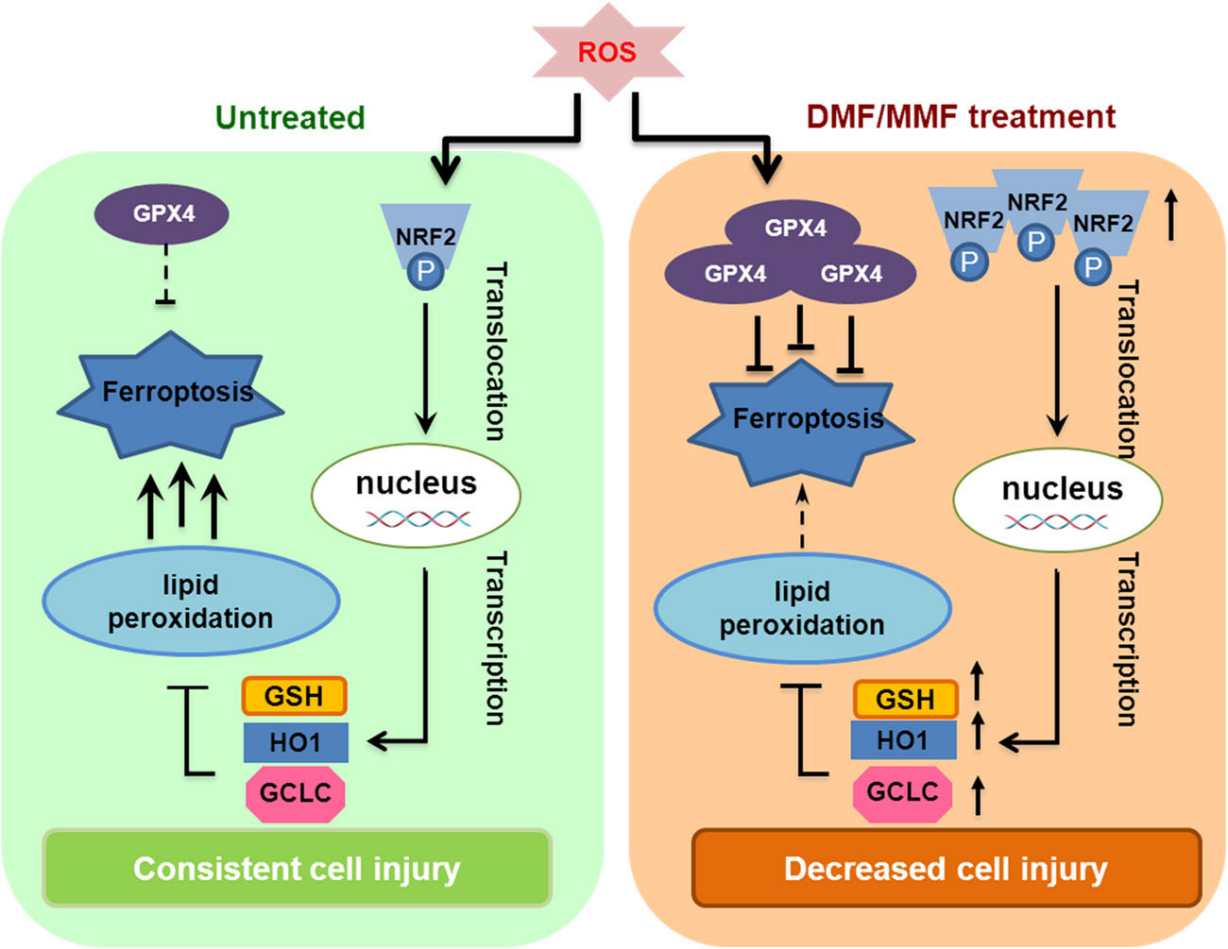
Working model. Shown is a working model depicting how fumarates protect liver cells from ROS damage by activating the NRF2 antioxidant pathway and inhibiting ROS-induced lipid peroxidation and ferroptosis. Fumarates increase the activity of NRF2, and upregulate the protein levels of NRF2, GCLC, HO1, and GPX4, thereby leading to a decreased ROS level, cell lipid peroxidation, and ferroptosis.

Ferroptosis is an iron- and ROS-dependent form of cell death, which is characterized by the accumulation of lipid hydroperoxides to lethal levels^36,37^. ROS accumulation could directly react with unsaturated fatty acids, which may lead to a destruction of the mitochondrial membrane, a massive release of substances promoting apoptosis, and increased ferroptosis. Dysregulation of ferroptosis has been implicated in various pathological processes, including cancer, neurodegenerative diseases, acute renal failure, drug-induced hepatotoxicity, ischemia reperfusion injury, and T-cell immunity^38,39^. Our study showed that fumarates upregulate the protein level of GPX4, a GSH-dependent enzyme that reduces lipid hydroperoxides, while decrease lipid peroxidation and ferroptosis, and thus ameliorate ethanol-induced liver injury in the ALD mouse model (Fig. 6). In addition, these findings support that fumarates could also be effective in other ferroptosis-associated diseases.

In recent years, drug repurposing has gained more and more attention for accelerating drug development^40^. Given the high costs, possible side effects, high failure rate and long testing periods for developing new medicines^7^, drug repurposing provides an attractive approach to meet the need for improved diseases treatment. For example, disulfiram, an old alcohol-aversion drug, has emerged as a candidate for treating high-risk breast cancer^7^. Hippeastrine hydrobromide (HH), which has been used to prevent avian influenza H5N1, has become a promising drug for inhibiting Zika virus (ZIKV)-infection^41^. Topiramate, a safe and effective drug for treating neurological diseases, is capable of ameliorating inflammatory bowel disease^42^. In this study, we demonstrate that computational repositioning of FDA-approved drugs by analyzing public gene-expression data can be used to infer drug therapies for ALD, and offer experimental evidence that the NRF2 inducer DMF is capable of ameliorating disease pathophysiology in the ALD mouse model. DMF was already established as a safe and effective drug for treating multiple sclerosis^43^. Additional clinical investigation will be needed to test whether DMF could benefit patients suffering from ALD.

## Materials and methods

### Cell culture and treatment

Cell culture was performed as previously described^44^. HepG2 or LO2 cells were cultured in DMEM/high glucose medium (HyClone, SH30022.01) or RPMI medium modified (HyClone, SH30809.01) supplemented with 10% fetal bovine serum (Gibco, 10091148), 1% penicillin and streptomycin (Gibco, 10378016) at 37 °C, in a humidified atmosphere containing 5% CO_2_. For fumarate treatment, cells were first cultured in the medium which contained fetal bovine serum. Then DMF (Sigma-Aldrich, 242926) and MMF (Sigma-Aldrich, 651419) of different concentrations were added into the medium. The treatments to increase cell oxidative stress and ferroptosis were performed by adding ethanol (Sigma-Aldrich, E7023; 80 mM), doxorubicin/DOX (Solarbio, D8740; 10 μM), and erasitin (Selleck, S7242; 10 μM) to the culture medium for 6 h, then we treated liver cells with fumarates or ferrostatin-1 (Sigma-Aldric, SML0583; 1 μM) for another 6 h; all the concentrations are final concentrations in the culture medium.

### Western blotting

Western blotting was performed as previously mentioned^45,46^. HepG2 or LO2 cells were lysed in RIPA lysis buffer (Beyotime, P0013B) containing protease and phosphatase inhibitors. Cell debris was removed by centrifugation while cell lysates were boiled for 10 min and centrifuged at 4 °C before loading on 10% or 12% SDS-PAGE gels. Then proteins were transferred onto PVDF membranes (Merck Millipore Ltd. IPVH00010) for western blotting analysis. The primary antibodies to phosphor-S40 NRF2 (Abcam, ab76026; 1:10,000 working dilution), NRF2 (Proteintech, 16396-1-AP; 1:1000 working dilution), GCLC (Proteintech, 12601-1-AP; 1:500 working dilution), HO1 (Proteintech, 10701-1-AP; 1:1000 working dilution), α-tubulin (Proteintech, 66031-1-lg; 1:1000 working dilution), GXP4 (Abcam, ab125066; 1:1000 working dilution), Histone-3 (Proteintech, 17168-1-AP; 1:1000 working dilution), ACTB/β-actin (Proteintech, 20536-1-AP; 1:1000 working dilution) were commercially obtained.

### RNA interference

Knocking down of *NRF2* was performed by RNA interference, following the manufacturer’s instructions for Lipofectamine RNAiMAX reagent (Invitrogen, 1875254). The knockdown efficiency was determined by western blotting. Synthetic siRNA oligo nucleotides were obtained commercially from Genepharma Co, Ltd. List of effective sequences is as follows: si*NRF2*-1: 5′-GGUUGAGACUACCAUGGUUTT-3′

si*NRF2*-2: 5′-CCAGAACACUCAGUGGAAUTT-3′

si*NRF2*-3: 5′-GCCUGUAAGUCCUGGUCAUTT-3′

Negative control: 5′-UUCUCCGAACGUGUCACGUTT-3′

### Cytoplasmic and nuclear extracts

For NRF2 nuclear translocation experiments, cells were cultured in the medium which contained fetal bovine serum, then DMF and MMF of different concentrations were added into the medium for 6 h. In all, 10-cm-diameter plates of HepG2 and LO2 cells were lysed, and cytosolic and nuclear fractions were separated following the protocol provided by the nuclear and cytoplasmic extraction kit manufacturer (Active Motif Inc, 40010). The nuclear pellets were washed three times with phosphate buffered saline containing freshly added protease and phosphatase inhibitors. The cytosolic supernatant was centrifuged to remove any nuclear contamination and transferred to a new tube. Both the cytosolic and nuclear fractions were boiled separately in SDS sample buffer, and analyzed by western blot.

### GSH analysis

HepG2 cells were plated into white and flat-bottom 96-well plates, and cultured for 4 h at 37 °C. Then we treated cells with DMSO or fumarates, and incubated for another 20 h. For fluorescent GSH assay, we first washed cells with Hanks’ balanced salt solution (Solarbio, H1045-500), then determined the levels of reduced and oxidized GSH by GSH/GSSG Assay kit (Promega, V6611), according to the manufacturer’s protocol. Total relative luminescence units (RLU) are graphed as means ± SD. *denotes *P* < 0.05, NS denotes no significance. Graphed data represents one of three experimental repeats.

### Measurement of cell lipid peroxidation and NADP/NADPH assay

Liver cells were plated into 60-mm dishes, and cultured for 24 h at 37 °C. The treatments to increase cell lipid peroxidation were performed by adding ethanol (80 mM), doxorubicin/DOX (10 μM), and erasitin (10 μM) to the culture medium for 6 h, then we treated liver cells with or without fumarates erasitin (10 μM) or ferrostatin-1 (1 μM) for another 6 h; all the concentrations are final concentrations in the culture medium. For lipid-peroxidation assay and NADP/NADPH assay, we first washed cells with 4 °C precooled phosphate buffered saline, then determined the levels of cell lipid peroxidation by MDA assay kit (Beyotime, S0131) and NADP/NADPH quantitation colorimetric kit (BioVision, K347), according to the manufacturer’s protocol. The total hepatic MDA content and NADP/NADPH levels are graphed as means ± SD. Graphed data represent one of three experimental repeats.

### Immunofluorescence staining

HepG2 cells were plated into glass bottom cell culture dishes (NEST, 801001) and pre-treated with or without DOX for 6 h, followed by addition of DMF and MMF into the medium. Thereafter cells were first fixed with 4% paraformaldehyde (Biosharp, 1707182), then permeabilized in 0.2% Triton X-100 (Amresco, 0694), blocked by 5% bovine serum albumin (Amresco, 0332) in PBS buffer (Sigma-Aldrich, P5368), and lastly incubated with the indicated primary NRF2 antibody (1:200 working dilution), which corresponds with fluorescent-conjugated secondary antibody.

For cellular ROS-level assay, HepG2 cells were treated as indicated, then loaded with DCFH-DA (Sigma-Aldrich, D6883; 10 μM) and incubated for 30 min at 37 °C in the dark after washing with Hanks’ balanced salt solution. All the fluorescence images were obtained with the laser-scanning confocal microscope. The relative NRF2 and phosphorylated NRF2 localization in nuclei were calculated with ImageJ software.

For lipid-peroxidation-level assay, LO2 cells in glass bottom culture dishes were incubated by BODIPY^®^ 581/591 C11 lipid-peroxidation sensor (Invitrogen, D3861). Stock solution in ethanol was diluted with PBS buffer to a final concentration of 4 μM. Cells were stained for 30 min in darkness to avoid accelerated oxidation. Then, LO2 cells were washed three times and observed with the fluorescence microscope. The fluorescence of BODIPY^®^ 581/591 C11 was acquired simultaneously using dual excitation (485 and 581 nm) and detection (510 and 591 nm).

### ALD mouse model

For the ALD mouse model, 8-week-old C57BL/6 male mice with body weight over 20 g were acquired from the Experimental Animal Department of Central South University and maintained in ventilated cages under specific pathogen-free conditions at the animal laboratory of Xiangya medical school. The procedures related to mouse subjects were approved by the Ethics Committee on animal research of Xiangya Hospital, Central South University. We divided C57BL/6 mice into four groups of six, and fed them with a standard Lieber-DeCarli liquid diet (Bio-Serv Company, England) for 5 days, followed by a liquid ethanol diet or control diet for 10 days, respectively. From the 11th day morning, the mice were given DMF (100 mg/kg and 200 mg/kg, respectively) or maltodextrin (control) by oral gavage and sacrificed after 10 days^47^.

### Histology, IHC, and transmission electron microscopy

Liver tissues from animal models were fixed in 10% buffered formalin for 8 h, followed by transfer to 70% ethanol, then embedded by paraffin. Sectioned liver tissues (5 μm) were stained by H&E according to the manufacturer’s protocol. For IHC staining, we performed the experiment as described^48,49^. The primary antibodies to NRF2 (1:200 working dilution), GCLC (1:100 working dilution), GXP4 (1:100 working dilution), and 4-HNE (Abcam, ab46545; 1: 100 working dilution) were commercially obtained. Negative control slides were performed without primary antibody. Control slides known to be positive for each antibody were incorporated. To quantify the IHC result of positive staining, the tissue areas of five ducts (173 mm^2^) in each sample were microscopically examined and analyzed by an experienced pathologist. Liver tissues (1 × 1 × 1 mm) were fixed by glutaraldehyde, and observed under transmission electron microscopy (FEI, Hillsboro, USA) at the Electron Microscopy Core Facility, Xiangya Hospital, Central South University. Images were captured using a charge-coupled device camera and analyzed using Motic Images Advanced software.

### Statistical analysis

All values included in the figures represent mean ± SD. Error bars represent ± SD for triplicate experiments. The statistical significance is indicated as asterisks (*). Two-sided *P* value of <0.05 was considered to be statistically significant (*P < 0.05, **P < 0.01, ***P < 0.001).

## Supplementary information

supplementary file
