# Supplementary material for: Computational repositioning of dimethyl fumarate for treating alcoholic liver disease
Source: Cell Death Dis. 2020 Aug 18;11(8):641. doi: 10.1038/s41419-020-02890-3 (PMC7434920; doi:10.1038/s41419-020-02890-3)
Supplement: Supplementary file 1 — supplementary file [file 41419_2020_2890_MOESM1_ESM.docx]

## SUPPLEMENTAL INFORMATION

**Computational repositioning of dimethyl fumarate for treating alcoholic liver disease**

**Ye Zhang^1#^,Shuang Zhao^1#^,** Ying Fu^1^, Lu Yan^2^, Yilu Feng^1^, Yaqi Chen^2^, Yijia Wu^2^, Yalan Deng^1,2^, Guiying Zhang^2^, Zhuchu Chen^1^, Ting Liu^2*^, Yongheng Chen^1*^

**Inventory of Supplemental Information**

**Supplementary Figure 1: Linked to Figure 1**

**Supplementary Figure 2: Linked to Figure 2**

**Supplementary Figure 3: Linked to Figure 3**

**Supplementary Figure 4: Linked to Figure 4**

**
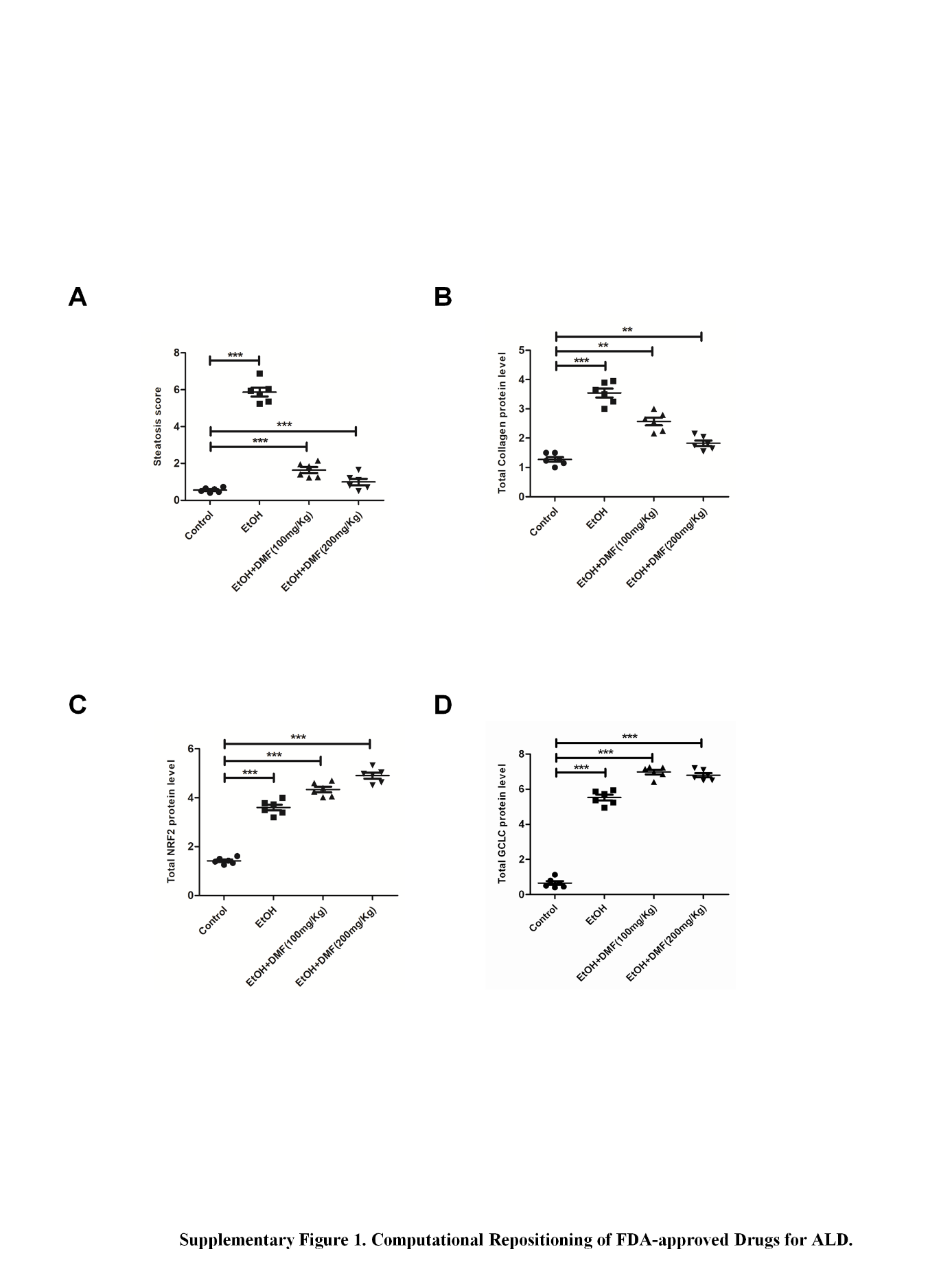
**

**Supplementary Figure 1. Computational Repositioning of FDA-approved Drugs for ALD. (A)** Hematoxylin and eosin (H&E) staining quantification in mouse liver tissues. The intensities of hepatic steatosis in each group were quantified using the Motic Images Advanced software, followed by statistical analysis. **(B-D)** Immunohistochemical staining of Collagen, NRF2 and GCLC proteins in mouse liver tissues. The statistical analysis of all liver tissue sections stained with anti-Collagen antibody **(B)**, anti-NRF2 antibody **(C)** or anti-GCLC antibody **(D)** was shown. The symbol * showed statistically significant differences with *p < 0.05, **p< 0.01 and ***p< 0.001.

**
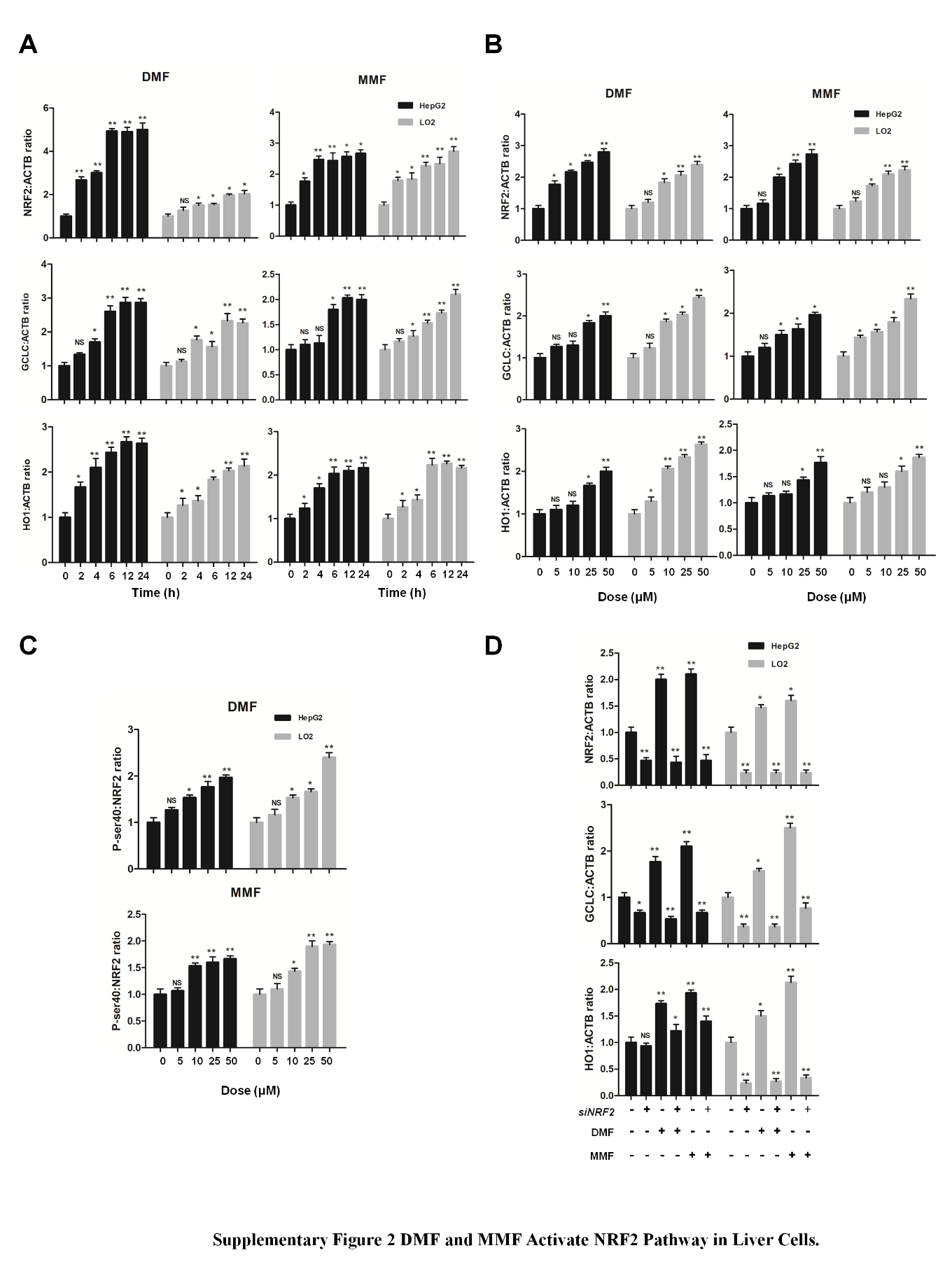
**

**Supplementary Figure S2. DMF and MMF Activate NRF2 Pathway in Liver Cells. (A)** DMF or MMF treatment increases endogenous NRF2, GCLC and HO1 protein level in a time-dependent manner. The relative NRF2, GCLC and HO1 protein compared with ACTB level were quantified respectively. The symbol * showed statistically significant differences with *p < 0.05, and **p< 0.01, NS, no significance. Error bars represent ± S.D. for triplicate experiments. **(B)** DMF or MMF treatment increases endogenous NRF2, GCLC and HO1 protein level in a dose-dependent manner. The relative NRF2, GCLC and HO1 protein compared with ACTB level were quantified respectively. The symbol * showed statistically significant differences with *p < 0.05, **p< 0.01 and NS, no significance. Error bars represent ± S.D. for triplicate experiments. **(C)** DMF or MMF increases NRF2 S40 phosphorylation level. The relative NRF2 S40 phosphorylation compared with its total protein level was quantified. The symbol * showed statistically significant differences with *p < 0.05, **p< 0.01 and NS, no significance. Error bars represent ± S.D. for triplicate experiments. **(D)** *NRF2* knockdown decreases GCLC and HO1 protein levels under normal or fumarates condition. The relative NRF2, GCLC and HO1 protein compared with ACTB protein were quantified respectively. The symbol * showed statistically significant differences with *p < 0.05, **p< 0.01 and NS, no significance. Error bars represent ± S.D. for triplicate experiments.


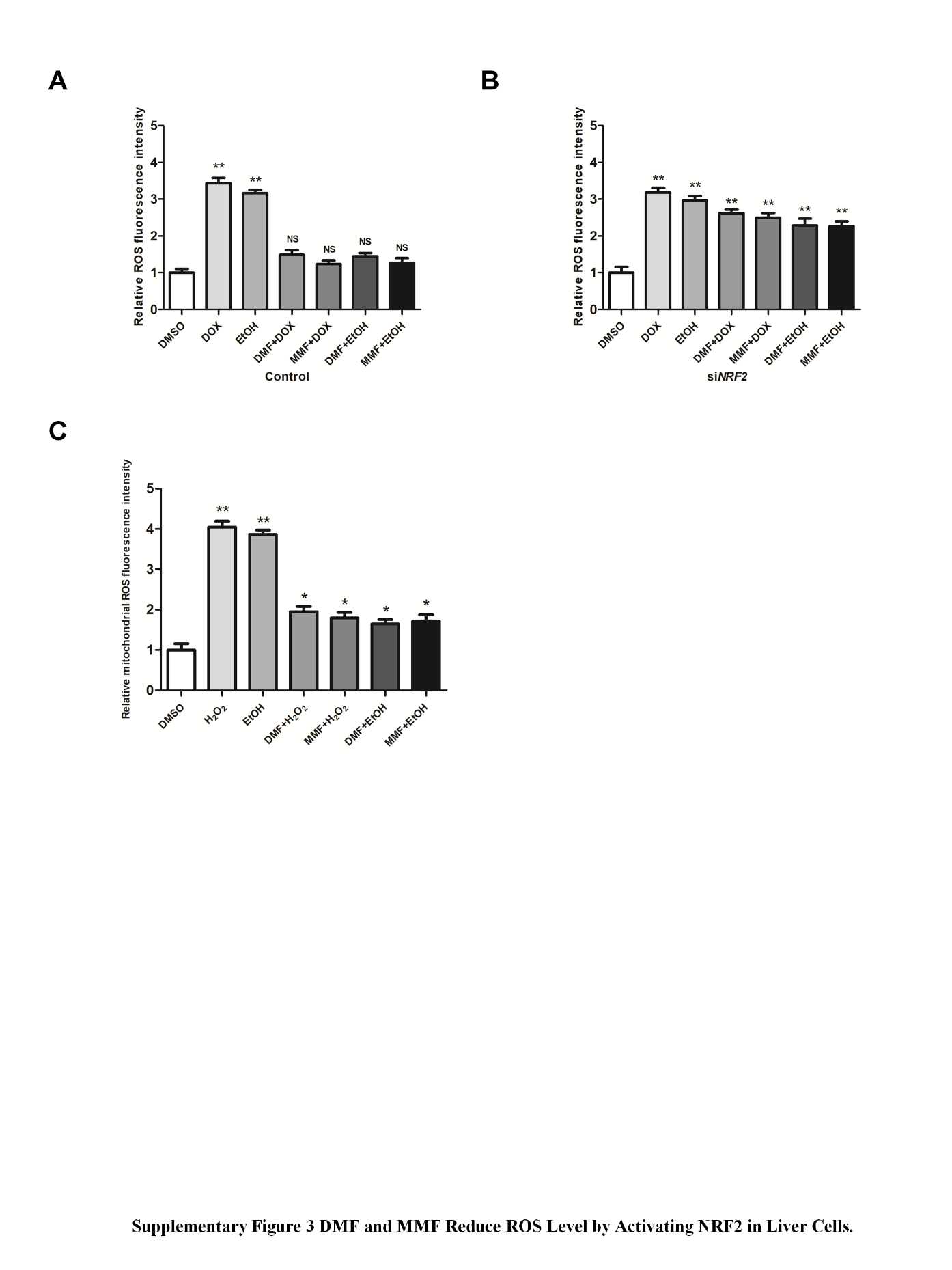


**Supplementary Figure S3. DMF and MMF Reduce ROS Level by Activating NRF2 in Liver Cells. (A and B)** Fumarates blocked DOX or ethanol-induced ROS accumulation. Relative ROS accumulation without **(A)** or with **(B)** NRF2 knockdown was calculated using ImageJ software; the ratio was quantified. The symbol * showed statistically significant differences with *p < 0.05, **p< 0.01 and NS, no significance. Mean values were calculated from the individual distributions in 10 cells per condition. **(C)** Fumarates reduced H_2_O_2_ or ethanol-induced mitochondrial ROS. Relative mitochondrial ROS fluorescence intensity under different treatment was quantified, normalized to the DMSO group. *p < 0.05, and **p< 0.01 were based on the Student’s t test. Error bars represent ± S.D. for triplicate experiments.

**
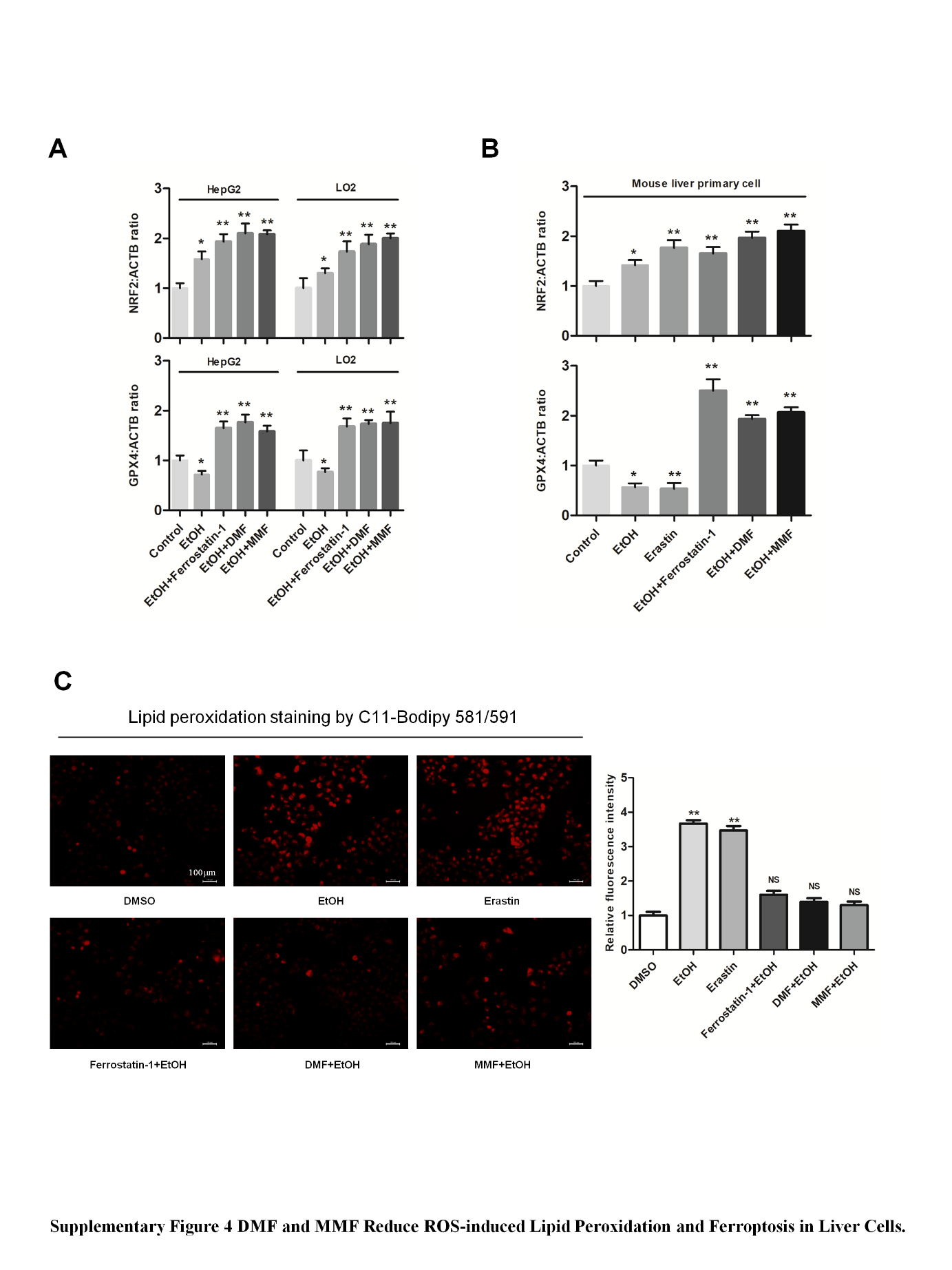
**

**Supplementary Figure S4. DMF and MMF Reduce ROS-induced Lipid Peroxidation and Ferroptosis in Liver Cells. (A and B)** Fumarates block lipid peroxidation and ferroptosis in liver cells. The relative NRF2 and GPX4 protein compared with ACTB in HepG2 cells, LO2 cells **(A)** or mouse liver primary cells **(B)** were quantified respectively. The symbol * showed statistically significant differences with *p < 0.05, **p< 0.01. Error bars represent ± S.D. for triplicate experiments. **(C)** Fluorescence microscopy of lipid peroxidation (using C11-BODIPY 581/591). LO2 cells were pre-treated with ethanol or erastin for 6 h, followed by treatment with 1 μM ferrostatin-1 or 10 μM fumarates for another 6 h as indicated. Cells were loaded with C11-BODIPY 581/591 and incubated for 30 min at 37 °C in the dark. Images were acquired by fluorescence microscope. Bar indicates 100 μm. Relative fluorescence intensity was calculated using ImageJ software; the ratio was quantified.
